# Digital Image Analysis of the Angle and Optic Nerve: A Simple, Fast, and Low-Cost Method for Glaucoma Assessment

**DOI:** 10.1155/2020/3595610

**Published:** 2020-10-28

**Authors:** Greg Russell, Silvia N. W. Hertzberg, Natalia Anisimova, Natalia Gavrilova, Beáta É. Petrovski, Goran Petrovski

**Affiliations:** ^1^Eyenuk Inc., Clinical Development, Woodland Hills, CA, USA; ^2^University of Szeged, Department of Ophthalmology, Szeged, Hungary; ^3^Center for Eye Research, Department of Ophthalmology, Oslo University Hospital, Institute for Clinical Medicine, Faculty of Medicine, University of Oslo, Oslo, Norway; ^4^The A. I. Evdokimov Моscow State University of Medicine and Dentistry of the Ministry of Healthcare the Russian Federation, Moscow, Russia; ^5^Eye Center “Vostok-Prozrenie”, Moscow, Russia; ^6^Faculty of Dentistry, University of Oslo, Oslo, Norway

## Abstract

**Purpose:**

To devise a simple, fast, and low-cost method for glaucoma assessment using digital image analysis of the angle and optic nerve in human subjects.

**Methods:**

Images from glaucoma and fundus assessment were used in this study, including color fundus photographs, standard optic nerve optical coherence tomography (OCT), and digital slit-lamp images of the angle/gonioscopy. Digital image conversion and analysis of the angle using ImageJ (NIH, USA) and adaptive histogram equalization contrast-limited AHE (CLAHE) to prevent noise amplification were implemented. Angle and optic nerve images were analyzed separately in the red, green, and blue (RGB) channels followed by 3D volumetric analysis of the degrees of angle depth and cup volume of the optic nerve. Horizontal tomogram reconstitution and nerve fiber detection methods were developed and compared to standard OCT images.

**Results:**

Digital slit-lamp angle images showed similar accuracy as standard anterior OCT measurements. Comparative analysis of RGB channels produced volumetric cup and horizontal tomogram, which closely resembled the 3D OCT appearance and *B*-scan of the cup, respectively. RGB channel splitting and image subtraction produced a map closely resembling that of the retinal nerve fiber layer (RNFL) thickness map on OCT.

**Conclusions:**

While OCT imaging is rapidly progressing in the area of optic disc and chamber angle assessment, rising healthcare costs and lack of availability of the technology open a demand for alternative and cost-minimizing forms of image analysis in glaucoma. Volumetric, geometric, and segmentational data obtained through digital image analysis correspond well to those obtained by OCT imaging.

## 1. Introduction

The need for improved management of glaucoma seems obvious with the increasing prevalence worldwide. A recent study correctly predicted that a total of 79.6 million people will be affected by glaucoma in 2020, which is indeed the case today. Out of these, bilateral blindness was estimated to occur in 11.2 million [1]. The following 20 years might bring rise in the number of glaucoma patients up to 111.8 million [2]. While the prevalence of glaucoma has a societal burden, there is an associated economic burden, which affects both developing and developed countries with at least the United States spending approximately $6 billion in 2013; the latter accounted for productivity losses and individual patient care per year [3]. In the United Kingdom, medical costs were approximately over 40% of the direct costs [4]. In sub-Saharan African countries, the lowest income patients diagnosed with glaucoma can spend almost 100% of their salary on treatment, while middle-income earners spent at least 50% of their salary on the disease treatment [5, 6].

Glaucoma is classified as a group of diseases with progressive damage to the optic nerve. It can be divided into primary open angle glaucoma (POAG), primary angle-closure glaucoma (PACG), and secondary glaucoma. The most recognized risk factor for glaucomatous progression is increased intraocular pressure (IOP), which occurs in eyes with an imbalance between aqueous liquor production and drainage through the angle. In this matter, the angle drainage becomes essential, and angle anatomy is doubtlessly an important factor for assessment in glaucoma management. Worldwide, the prevalence of POAG shows variation, which can be attributed to multiple factors including ethnicity. Meta-analysis reveals a prevalence of POAG of 1.4% in Asian, 2.0% in Caucasian, and 4.2% in the African population [7]. In contrast, PACG occurs more frequently in Asia than in Europe with estimates of 1.5% and 0.04%, respectively [8, 9].

The burden of blindness from glaucoma is challenged not only by the increasing number of cases but also by the difficulties to recognize the affected population. Glaucoma is an asymptomatic disease, which only raises suspicion when it becomes advanced. Studies have estimated the prevalence of undetected glaucoma to be 50% in developed countries and up to a striking 98.5% in developing countries [10–12]. On the other hand, approximately half of the patients diagnosed and treated with glaucoma are found to be ocular hypertensives that may not be benefiting from treatment at all [11, 13, 14].

The notable number of undetected glaucoma cases and overtreated patients has given rise to multiple evaluations of screening tools to trace unrecognized glaucoma. Yet, the United States Preventive Services Task Force (USPSTF) has indicated a lack of convincing evidence to facilitate regular screening, whereas no screening tool has been shown cost-beneficial to date [15, 16]. The current effort focuses on how to manage and follow the increasing number of patients with glaucoma [15]. Nevertheless, there is an increasing demand for organized screening programs that are cost-effective to prevent the load of future visual impairment in glaucoma patients, similar to diabetic retinopathy screening [10, 12, 15].

In addition to the increasing burden of glaucoma, the imminent shortage of healthcare professionals worldwide strengthens the need for the development of cost-beneficial screening methods [17, 18]. In this matter, emphasis on office visits may shift to alternative methods with reading centers or even automated image analysis. While noninvasive optical coherence tomography (OCT) is rapidly progressing in means of optic disc and chamber angle assessment and is considered a standard in angle-closure detection compared to gonioscopy [19], with rising healthcare costs, demand and availability for the technology can split, leaving lower-financed practices short of aid. Other simple, fast, and low-cost forms of image analysis need to be incorporated to help in the management of glaucoma or to preassess those attending for care. The sharp rise in the number of smartphones with high resolution cameras worldwide poses a potential source of documentation, storage, sharing, and eventually image analysis of large numbers of photographs taken to be used in ophthalmic decision-making [20–23]. Though, at present, image acquisition and interpretation are still having many limitations, it seems reasonable to develop methods and algorithms that can assess a mass of people at an affordable price to allocate further attention to those who are in need of medical care. In this pilot study, we present the information that is to be gained from color photographs of the fundus and the chamber angle, regarding glaucoma. Volumetric, geometric, and segmentational data are gathered and compared with corresponding high-definition OCT images to test for comparison and possible validity, while the cost-benefits of such processing are shown as well.

## 2. Methods

Images from glaucoma and fundus assessment were used in this study, including freely available/online color fundus photographs, standard optic nerve optical coherence tomography (OCT), and additional digital slit-lamp images of the angle obtained by gonioscopy (no patient contact was needed for the image analysis, and the whole process was carried out without knowing any personal data for the images included in the processing). Applying the diagnostic criteria for glaucoma, a novel method is hereby aimed at showing similarities and possible data correlation between findings, data presented by *B*-scans, and the provided standard grade for glaucoma.

### 2.1. Contrast Enhancement

Standardization of the fundus images was achieved through known methods of image processing by ImageJ (NIH, USA). Normalization of the histographic information across each image was achieved as volumetric representation and was derived from image intensity. As image intensity varied according to camera exposures, histogram equalization (HE) was needed to standardize the images. In addition, comparative to HE, contrast-limited adaptive histogram equalization (CLAHE), a tool designed to prevent the over amplification of noise that adaptive HE can give rise to, was used in the study. Splitting of the color fundus images into red, green, and blue (RGB) channels was performed to achieve optimal comparison and dynamic range of the images acquired.

### 2.2. 3D Volumetric Measurement of Image Intensity and Analysis of the Profile of the Cup

After application of CLAHE in the regions of interest (ROI), the images were converted into a 3D representation in ImageJ by adjusting volumetric measurements of the image intensity through a 3D rendering on the screen and an interactive 3D-surface plot function. Splitting of the RGB was carried out consequently, and the optimal channel was selected for further analysis. Images taken from the fundus were imported into ImageJ for further analysis.

### 2.3. Retinal Nerve Fiber Layer (RNFL) Detection Method

Images were split into RGB, and then, the differences between the channels were observed, and a spectrum lookup table was applied to allow image intensities to be compared to the gold standard images and data produced by the RNFL analysis given by the spectral domain Topcon 3D-2000 OCT. RGB fundus imaging separates a fundus image into three wavelength components to help visualize the retinal pigment epithelium/choroid (red filter), neural retina (green filter), and nerve fiber layer (blue filter). This allows for better visualization of the retinal tissues [24].

## 3. Results

### 3.1. Contrast Enhancement

**Figure 1:**
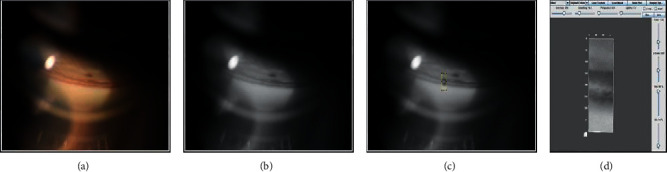
Green channel of a gonioscopy image used for ROI selection and consequent CLAHE filtering. (a) Gonioscopy image. (b) 8-bit converted image. (c) Selection of area of interest. (d) Interactive 3D-surface plot.

RGB color splitting allowed for a more averaged distribution of the intensity range across the ROI selected by the user (Figure 1). The color fundus and gonioscopy images were split into RGB channels to allow for expert observation and comparison of findings. The green channel was found to show the highest dynamic range for the anterior images and was used to further select the ROI to be processed with the CLAHE filter.

### 3.2. 3D Volumetric Measurement of Image Intensity in the Angle

**Figure 2:**
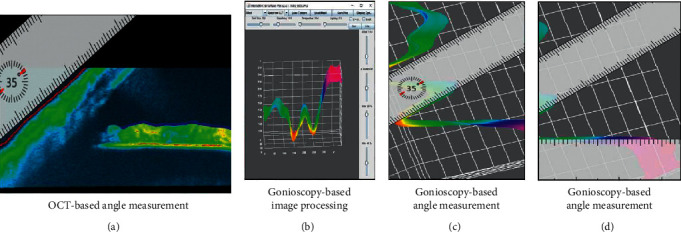
3D measurement of the image intensity and angle from gonioscopy images in comparison to angle measurements obtained by anterior OCT. A gonioscopy-based image processing includes intensity adjustment and clipping of the intensity range, followed by angle measurement; for comparison, the corresponding angle obtained by anterior OCT measurement is shown. (a) OCT-based angle measurement. (b) Gonioscopy-based image processing. (c) Gonioscopy-based angle measurement. (d) Gonioscopy-based angle measurement.

Parameters in the screen were adjusted to the range as shown in Figure 2 for the gonioscopy images before any adjustments, to avoid clipping of the intensity range, and then, the angle was calculated as shown. The detected angle was then compared to the angle shown from the anterior OCT scan.

### 3.3. 3D Volumetric Measurement of Image Intensity of the Fundus and Analysis of the Cup Profile

**Figure 3:**
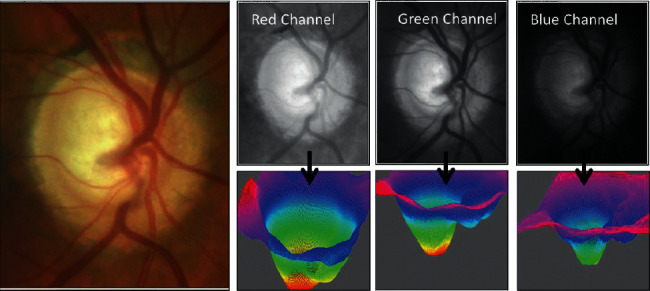
Splitting of the RGB channels in fundus images of the optic nerve and 3D volumetric conversion.

The review of split RGB channels concluded that the green channel harvested better information (Figure 1). However, when applied to the fundus images and the optic nerve (Figure 3), the red channels offered a less-obstructed view of the cup region of the images compared to the blue or green channel. It was considered important here for the dynamic range to support analysis of the cup volume through the profile shown.

**Figure 4:**
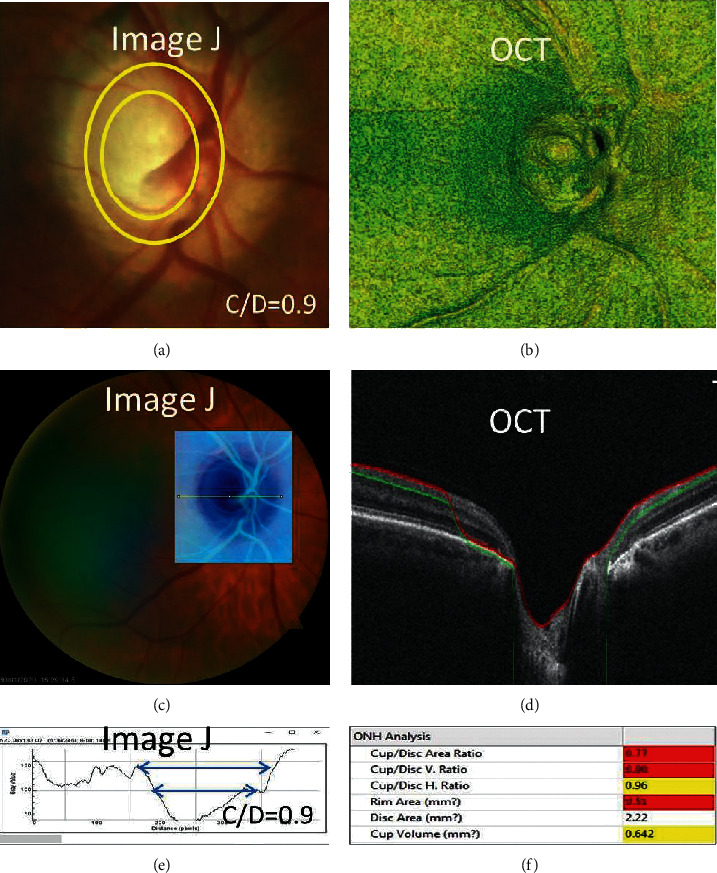
Resemblance of the ImageJ histogram and cup/disc (C/D) ratio based on the 3D fundus images compared to the analogous images obtained by OCT.

The images, both by profile and when rendered in 3D, appeared to show a close resemblance to the tomographical images shown in Figure 4.

### 3.4. RNFL Detection

**Figure 5:**
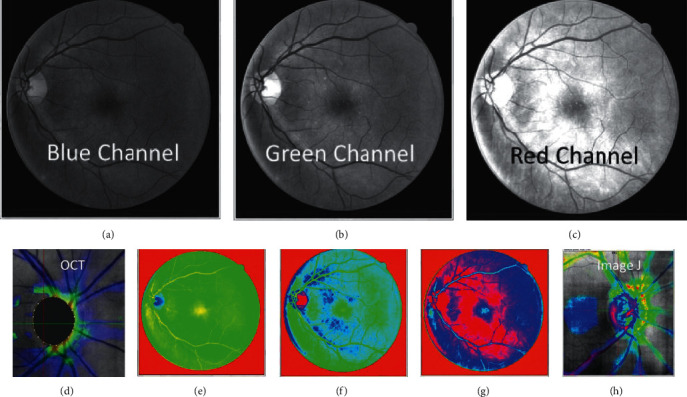
Splitting of the RGB channels in fundus images/optic nerve and resemblance of the blue channel ImageJ to the OCT thickness map of the RNFL.

Splitting of the RGB channels in the fundus/optic nerve images (Figure 5) and obtaining differential images between the color channels resulted in the blue channel showing similar intensity when analyzed by ImageJ as the Topcon 3D OCT channel shown in the same figure.

## 4. Discussion

### 4.1. Image Analysis Technique

Normalization of histographic information across angle images obtained from gonioscopy is important to standardize the image intensity across a dynamic range and across the image ROIs. The CLAHE filter can be applied initially to RGB split images to look for the best possible profile in the intensity images. Figure 5 illustrates the strength of the green channel, showing more dynamic range and resolving more information than the other color profiles do.

ImageJ splitting of fundus images into red, green, and blue color channels allows a simple way to obtain monochromatic renderings from any full color acquisitions; a disadvantage of doing so is that some loss in resolution can occur as a result of viewing just a single channel. Nevertheless, as exposure often needs to be increased to compensate for light loss when a physical filter is introduced into the light path of the fundus camera, this splitting technique allows for a more comfortable exposure for the patient when taking the image.

Green light is typically focused deeper into the retina, while red light is particularly good for visualizing the retinal pigment epithelium and the choroid, and blue light is good for determining the nerve fiber layer. Digital retinal photography is unique in comparison to film-based techniques; it allows for the immediate adjustment of exposure settings and offers easy contrast enhancement. The disadvantage to digital imaging is the linear response and narrow exposure latitude of currently available digital sensors. The problem of dynamic range has been acknowledged by a number of digital single lens reflex (DSLR) manufacturers; so, for example, some cameras have an automatic exposure bracketing mode that is used in conjunction with the high dynamic range imaging software. Some sensors such as the Fujifilm Super CCD combines sensors of different sizes to give increased dynamic range, while other manufacturers use in-camera software to prevent highlight overexposure such as the D-Lighting feature from Nikon.

Unlike the traditional single-lens reflect camera, screeners take images on a fundus camera where the exposure settings have been typically predetermined by the software for standardization. The only variable the screener has to mitigate is the limited dynamic range of digital sensors due to small pupil settings or the flash level of the fundus camera. This is especially important to consider when imaging the optic nerve because of the stark contrast between the nerve and the surrounding retina. Images can also suffer from degradation caused by media opacity, and assessability has to be considered before reading such images [25].

It is important to have proper exposure as well; over or under exposure can be detrimental to digital image quality, while the exposure control requires a delicate balance between flash output, sensor gain, and gamma settings. The International Standards Organization (ISO) has a standardized scale for measuring the sensitivity of the film to light. These standards have traditionally been used with film-based fundus cameras [26].

The 3D volumetric measurement of image intensity and analysis of the profile of the cup was made possible by applying CLAHE in the ROI and by conversion of the images into 3D representative images in ImageJ and adjusting the volumetric measurements of the image intensity through a 3D rendering on the screen and an interactive 3D-surface plot function. Splitting of the RGB allowed for selection of optimal channel for further analysis. Using multiple filters in a photo assay style approach, one can provide the examiner with more than one monochromatic interpretation of the fundus, as is the case of epiretinal membranes and macular pigmentary changes detected at 490 nm, 540 nm, and 615 nm wavelengths.

Using a modern retinal fundus camera, retinal ganglion axons can also be directly observed. The fine nerve fibers reflect when imaged [27] and allow implementation of a scoring system; furthermore, texture analysis for severe RNFL defects can be performed from such images [28]. The microtexture analysis of the RNFL in the grey level digitized photographs has been described before [29]; depending on the aperture of the pupil, the flash intensity settings, and presence of, if any, polarization or cross polarizing filters in the optical pathway, appropriate images for assessment of RNFL can be obtained. One would also need to ensure some degree of normalization to average out the color range, hence our use of the CLAHE contrast enhancement filter. In the present study, we made an attempt to ‘color' the nerve fiber layer with a lookup table to show spectral analysis and compare it to the OCT structural texture analysis. Under such conditions, the dynamic and tonal range is important to be able to see the finest differences within the visible color gamut. The justification for using the blue channel comes naturally from the optical properties of the blue filter, which corresponds to the absorption spectrum of rhodopsin, which is around 500 nm.

### 4.2. Possible Applications in Ophthalmology

Multiple examinations are used in conjunction for diagnosis of glaucoma. The lack of a definitive reference standard limits both their specificity and sensitivity. Furthermore, there is no evidence that any combination of tests is superior in terms of patient outcome or cost-effectiveness [30]. Glaucoma is most frequently diagnosed via opportunistic case findings. Of all parameters that are taken into consideration, many practitioners are still relying heavily on IOP measurements, while disc assessment alone with or without visual field damage is underrepresented [31]. Findings suggest that variation amongst observers is large, and the rate of improper diagnosis is high, with more than half of glaucoma patients being missed on former ophthalmic visits [32]. With glaucoma being an optic neuropathy with functional loss occurring at later stages [33], it would seem reasonable to give emphasis to structural changes first, if one were to detect those at risk of a debilitating future visual disturbance and allocate further appointments to specialist care accordingly. Because the quality of life can be severely impaired if the disease is advanced bilaterally [34], selected cases require further attention. Current experience in glaucoma screening is not promising. The range of sensitivity and specificity for tests is large, while the study design, execution, and examined populations differ greatly. Though some tests may outperform others, no combination suitable for implementation to the general population has been established [35]. Identifying the population at risk may pose some challenges. Performing a community-based mass screening in an office setting has high financial, temporal, and professional requirements, thus not suitable for health systems already lacking funding. Narrowing of the target population is highly desired. Prepublicizing the risk factors for glaucoma does not seem to increase the number of patients successfully screened, for in self-recruited screening, overall health anxiety may surpass the actual rate of risk factors present [36]. Several studies suggest that there is a possible relationship between diabetes and POAG, the condition occurring almost twice as often in diabetics than in nondiabetics [37, 38]. It would seem plausible to use the large amount of fundus photos acquired during diabetic screening to devise and test an algorithm that can aid in the detection of optic disc pathologies, notably glaucoma. Additional costs for screening this way would be minimal, and a higher-than-normal prevalence of POAG might be anticipated. Furthermore, as individuals screened for any reason might have a false sense of security that they underwent a comprehensive eye test [39], screening for more than one condition could improve the hit ratio and lower the number of missed follow-up visits.

When making the diagnosis of glaucoma, physicians fail to properly complete gonioscopy about 50% of the time [40]. The true prevalence of PACG can easily be underestimated. Since all cases of glaucoma should be considered closed until confirmed otherwise [41], gonioscopy is an essential and compulsory tool for decision-making. PACG, although less frequent than POAG, leads to blindness more often; therefore, screening for narrow or closed angles would be worthwhile. Currently, only a few reports exist discussing the topic, mainly in well-defined Asian subgroups [8]. A popular method for detecting narrow angles, the Van Herick method for assessing the peripheral limbal depth, has been found to have a sensitivity and specificity of 61.9% and 89.3%, respectively [42]. Even with emerging OCT technology [19], 360^o^ visualization of the drainage structures is the best achieved by gonioscopy, which is still a gold standard in angle grading. To our current knowledge, total internal reflection from the chamber angle cannot be surpassed; otherwise, the need of a contact gonio lens appears to be a major weakness in designing a fast, infection-proof, noncontact, and reliable method suitable for screening. Nevertheless, image analysis of angle anatomy can be useful for future implications. Indeed, by applying the same contrast enhancement and volumetric measurement techniques described above on the temporal corneal periphery in photographs of the anterior segment, we can simulate the examination without the need of an additional light source for the slit beam.

Screening strategies for glaucoma with early diagnostic and prognostic value may decrease the societal cost in all regions of the world, especially among low-income earners. With the rising availability of less costly retina digital cameras, which cost 80% less to acquire than an OCT equipment itself [43, 44], a cost-minimizing image assessment system may lead to decreased costs and reduced individual burden associated to glaucoma such as fear of blindness, psychological health, and physical functioning due to eye sight loss. In addition, given the needed training in an optometry setting, such assessment can reduce the number of referrals for double screening, which takes on patient time, and which may also lead to disease progression. This will also provide a form of standardization for the rising concern about optometrists using gonioscopy in the primary care setting for glaucoma detection and the need for training nonophthalmic personnel in screening techniques, which can increase the sensitivity and specificity to a level of accurate positive prediction (62%) [45–48].

Altogether, we hereby present an alternative low-cost way to detect and manage glaucoma prospectively by applying a glaucoma assessment method using volumetric, geometric, and segmentational data obtained through digital image analysis, which correspond well to those obtained by high-definition OCT imaging. While the OCT technology is rapidly progressing in the area of optic disc and chamber angle assessment, rising healthcare costs and lack of availability of the technology open a demand for alternative forms of image analysis in glaucoma.

## Data Availability

The data used to support the findings of this study are available from the corresponding author upon request.
